# Suppression of Air Refractive Index Variations in High-Resolution Interferometry

**DOI:** 10.3390/s110807644

**Published:** 2011-08-02

**Authors:** Josef Lazar, Ondřej Číp, Martin Čížek, Jan Hrabina, Zdeněk Buchta

**Affiliations:** Institute of Scientific Instruments, Academy of Sciences of the Czech Republic, Královopolská 147, Brno 612 64, Czech Republic; E-Mails: ocip@isibrno.cz (O.C.); cizek@isibrno.cz (M.C.); shane@isibrno.cz (J.H.); buchta@isibrno.cz (Z.B.)

**Keywords:** refractometry, nanopositioning, interferometry, nanometrology

## Abstract

The influence of the refractive index of air has proven to be a major problem on the road to improvement of the uncertainty in interferometric displacement measurements. We propose an approach with two counter-measuring interferometers acting as a combination of tracking refractometer and a displacement interferometer referencing the wavelength of the laser source to a mechanical standard made of a material with ultra-low thermal expansion. This technique combines length measurement within a specified range with measurement of the refractive index fluctuations in one axis. Errors caused by different position of the interferometer laser beam and air sensors are thus eliminated. The method has been experimentally tested in comparison with the indirect measurement of the refractive index of air in a thermal controlled environment. Over a 1 K temperature range an agreement on the level of 5 × 10^−8^ has been achieved.

## 1. Introduction

Interferometric measuring techniques with a highly coherent laser source have become a cornerstone for measurement of geometrical quantities in primary metrology, calibration of mechanical length standards and also in industrial applications where ultimate precision is needed. The overall concept is based on a highly stable laser source with stabilized optical frequency representing a wavelength standard which is consequently seen as an elementary length counted by an interferometer. Further improvement of resolution of an interferometer below this length element has been achieved by a combination of optical techniques and advanced electronic digital signal processing of the interference signal. The stability of the optical frequency of laser sources which has been achieved recently is very high. Traditional He-Ne lasers stabilized to the active Doppler-broadened line in Ne can operate with relative frequency stability on the level 10^−8^–10^−9^, He-Ne laser stabilized through subdoppler spectroscopy in iodine on the 10^−11^–10^−12^ level and the potential of iodine stabilized lasers based on frequency doubled Nd:YAG is very close to the 10^−14^ level [1]. The reproducibility of their absolute frequencies is another goal in metrology and is limited to 2.1 × 10^−11^ and 9 × 10^−12^, respectively [2]. The conversion of a stable frequency into a precise wavelength relies on the value of speed of light which is a defined physical constant under vacuum conditions. In the laboratory environment the value of the refractive index of air has to be considered. The search and effort for a more precise interferometric measuring tool includes highly stable laser sources, reduction of noise, better optics, higher resolution through optical and electronic techniques, linearization, *etc.* [3–5]. Obviously, when measurement has to be performed in air—in the routine laboratory measurements—the refractive index of air represents a major source of uncertainty.

In a laboratory environment where calibrations and comparisons of interferometers are performed the use of an interferometric refractometer is necessary. The simplest configuration is a differential interferometer measuring with high resolution the difference between an air and vacuum path within the defined distance of an evacuated cell [6,7]; variations on this principle with heterodyne detection [8] or a semiconductor laser source [9] have been presented. Other approaches of refractometer design are trying to be compact and at the same time to find a more precise way of measurement where the value of refractive index is available on-line or at least more often than once the cell is evacuated and filled again. The systems include movable triangular cells, flexible cells that could be elongated [10], and some other arrangements [11–14]. Instruments designed to measure the refractive index on-line are called tracking refractometers; they should complement the most precise length measurements [15]. Tracking refractometer converting the refractive index variations into laser optical frequency has been presented in [16].

All measurements of the refractive index of air performed by refractometers or by evaluation of the Edlen formula suffer from one principal limitation namely the fluctuations of air along and around the laser beam axis. Furthermore, there are always thermal gradients present in the air—mainly in the vertical direction. The sensors, primarily thermal, can be placed close to the beam, but not, however, directly in the laser beam. Moreover, only selected points can be measured. The laser beam of the refractometer can be again placed only close to the measuring path. As a result evaluation of the refractive index of air through direct refractometery under laboratory conditions can be done with an uncertainty close to the 10^−9^ [8,17]. The most precise laboratory techniques seem to be those exploiting optical frequency comb synthesis [18–20]. The limiting factor seems to be the stability of the atmosphere around the beam path. The practical limit in evaluation of the refractive index of air is determined by effects such as thermal gradients and air fluctuations. They cannot be completely avoided; they depend on particular application and measurement configuration.

In case of all commercial interferometric systems the compensation of index of refraction of air is done by measuring of the fundamental atmospheric parameters—temperature, pressure and humidity of air, accompanied in some cases by the measurements of concentration of carbon dioxide. The value of refractive index is extracted by evaluation of the empirical Edlen formula [21]. This fundamental formula was further tested and a set of improvements followed [22–25]. Thus, the limits of this indirect determination of the refractive index are primarily given by the configuration of the measuring setup, by the air flow and stability of atmospheric conditions close to the laser beam rather than by the precision of sensors measuring temperature, *etc.* or the formula itself.

The fundamental parameters of atmosphere considered in the basic Edlen formula were temperature, pressure and humidity. A lot of effort has been put into evaluation of other effects such as content of various gasses in air, especially CO_2_ [26]. Humidity, particularly the content of water vapor has been investigated in [27,28]. The study of these effects resulted in inverse approaches where measurement of the refractive index of air became the means of determining of another quantity, such as temperature [29] or air density and moisture [30,31].

Efforts to combine the distance measuring interferometer and the refractometer into one instrument which could evaluate the influence of the refractive index of air during the measurement or directly compensate for it have been reported. There were arrangements presented where a complex set of two separate interferometers evaluates the refractive index of air and measures the distance [32]. This system can compensate for the refractive index, but is unable to overcome the problem of the determination of the refractive index in the laser beam axis. A method linking the wavelength of the laser source to the mechanical length of some frame or board was proposed in [33]. The authors suggested using a set of two identical interferometers where one is fixed in the length and serves as a reference for the laser wavelength. Other approaches represent completely different methods for determination of the refractive index of air, for example through the speed of sound at ultrasonic frequency range [34]. Also, the control of the refractive index which is kept constant was suggested [35].

## 2. Referencing of Wavelength

An interferometric system referenced to the stabilized laser needs a conversion to stable wavelength through the speed of light which includes a refractive index of the environment. The definition of the meter only involves the speed of light under vacuum, while most real measurements have to be performed in air. Also, commercial interferometers operate on air and their calibration must cover the compensation for varying wavelength caused by the environment.

**Figure 1.**
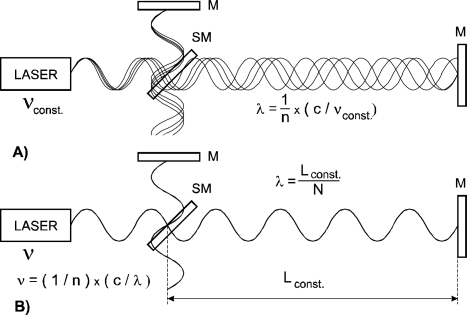
Varying wavelength in Michelson interferometer with laser with stabilized optical frequency (**A**) and interferometer with stabilized wavelength (**B**) M: mirror, S: semireflecting mirror, N: fixed number of wavelengths.

We propose an interferometric arrangement where the referencing could be derived not from a frequency and then transferred to wavelength via independently evaluated or measured refractive index of air, but from directly referencing the wavelength of the laser source. Fixing the laser wavelength over the measurement axis to a mechanical reference can replace a stabilization of the laser frequency. A He-Ne laser for interferometry locked to the active line in Ne can offer a relative stability of the optical frequency in the order of 10^−8^. This is on the same level as the coefficient of thermal expansion of highly stable materials such as Zerodur from Schott or ULE from Corning. It seems to be feasible to use mechanical standards made from these materials as a reference for stabilization of the wavelength under conditions of varying refractive index (Figure 1).

**Figure 2.**
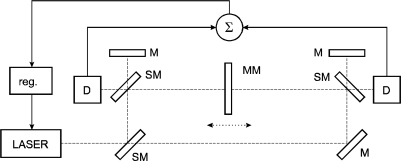
Principal schematics of the interferometric system with two countermeasuring Michelson Interferometers and stabilization of the laser wavelength to mechanical standard. D: detection unit, M: mirror, MM: movable mirror, SM: semireflecting mirror, reg.: regulator.

If displacement is measured, it must always be stated what is measured against what. All the traditional sources of error (refractive index, vibrations, laser noise, linearity, misalignment, *etc.*) can be marginal when there is a mechanical instability of the reference point. The approach we present here combines the mechanical referencing of the interferometer itself with referencing of the laser wavelength. The mechanical referencing simply cannot be avoided so we at least link one source of variations (refractive index) to another (mechanical). The use of a tracking refractometer with interferometric measurement may be a traditional approach. In our case these two devices merge together into one where it is not clearly divided which beam measures the refractive index of air and which one the displacement. From one point of view it may be a question which length out of the two measured ones in Figure 2 should be the correct one. From another one it is not possible to say, whether both are relevant or the stability of the frame is the key parameter. The solution presented here suggests measurement in a single axis which is the measurement axis both for evaluation of the refractive index of air and the measured length. There may be differences in refractive indices of the left and right measured paths (Figure 2) but expressing the resulting displacement as a mean value of these two reflects the concept that neither of these two can be seen as the “correct” one. In this case the refractometer and interferometer not only become one instrument, but the beam paths of these two are identical. This can be seen as the major advantage of this concept, reducing or even eliminating the sources of error arising from differences in measured refractive index and refractive index in the measuring path of the interferometer. When the wavelength (or mean wavelength) is fixed over the whole measuring range and the wavelength control loop is fast the system can adequately react even to fast changes of refractive index caused by turbulations or air flow. The question of dead length is not so relevant here while the stabilization of wavelength works in the whole range set by the position of the two interferometers. Obviously setting the range as short as needed is better and reduces the influence of variations along the beam path and between the left and right measured displacements.

The proposed arrangement in Figure 2 represents a combination of two counter-measuring Michelson interferometers where the position of the main beamsplitters and mirrors is fixed by a baseplate made of a low thermal expansion material. Constant length between the two reference points of the interferometers can be used to stabilize the wavelength of the common laser source through a constant integer number of wavelengths within the length and detection of a stable interference fringe.

## 3. Experimental Arrangement

To demonstrate the principle we assembled a setup with two flat mirror interferometers facing each other where the displacement measured by one is in the same axis measured by the other in opposite direction. Under stable conditions the sum value of the interferometer outputs should be constant. Both interferometers are supplied by a single laser source, in our case a tunable single-frequency He-Ne laser. The whole setup is fixed to a baseplate made of “0” grade Zerodur from Schott with a thermal expansion below 10^−8^/K in the temperature range from 0 °C to 50 °C. The thickness of the central moving mirror in the measuring arms of both interferometers contributes to the measured value, which is why it is also made from Zerodur.

The laser source used in our experiments allows fast PZT driven continuous mode-hop free tuning range up to 1.1 GHz of optical frequency. The laser is filled by a combination of isotopes of ^20^Ne and ^22^Ne, primarily to achieve a gain profile with a single gain maximum for a specific stabilization technique [36]. This causes an overall shift of the central lasing frequency which can be exploited here for easier measurement of laser frequency detuning without crossing the zero frequency. Tuning range of the laser source is here the limiting factor for covering the range of refractive index variations determined primarily by temperature. With a single-frequency He-Ne not more than 1 GHz can be covered, semiconductor lasers, such as those with external cavity can offer more [37–39].

**Figure 3.**
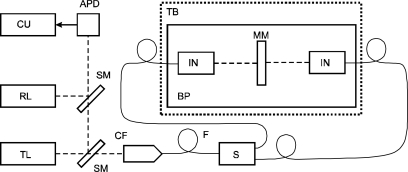
Arrangement of the interferometers with fiber-optic light delivery system monitoring laser frequency detuning by comparison with the reference laser. TL: tunable He-Ne laser, RL: reference stabilized He-Ne-I_2_ laser, CU: counter, APD: avalanche photodetector, SM: semireflecting mirror, CF: fiber collimator, F: single-mode polarization maintaining fiber, S: fiber splitter, TB: box with controlled temperature, MM: movable mirror, IN: interferometer, BP: baseplate.

In our arrangement the laser is locked to the zero sum output from the two interferometers and its frequency is tracked so that it can be compared with calculated values that follow variations of the refractive index independently evaluated through Edlen formula. The laser optical frequency is stabilized not to a frequency reference but to the mechanical one. Thanks to PZT tuning the control servo loop could be relatively fast with 1 kHz bandwidth. The laser amplitude noise produced fluctuations of the interferometers output below 1 nm level. The laser output is mixed with a reference He-Ne-I_2_ standard and the resulting beat frequency is recorded. A simplified schematic of the configuration is shown in Figure 3.

Control of the refractive index within this experiment was performed through an enclosure of the setup into a temperature controlled box with the possibility to control the temperature (Figure 3). The dependence of the refractive index of air on temperature is relatively significant, being 10^−6^/K. Moreover, the temperature can be very comfortably controlled. Our enclosure was designed as the double-walled glass box filled with water (water circulation is driven by a pump). We used water heating with the heat exchanger placed outside the box. The whole process was deliberately made slow (approx. 1 K/30 min,) in order to maintain an even distribution of the temperature. Heating through the walls together with complete enclosure suppressed thermal gradients in air and reduced air flow. A weather station monitoring temperature, pressure, humidity and content of CO_2_ was added with sensors placed in the box. Thermal control here was performed by a Peltier water/air thermal controller and heat exchanger able to control the temperature constant or to introduce controlled thermal drift. The six walls of the box were separate water containers with input and output, all interconnected by tubing where the design and sequence of water flow followed the demands for even distribution of temperature.

### 3.1. Temperature Compensated Interferometer Unit

**Figure 4.**
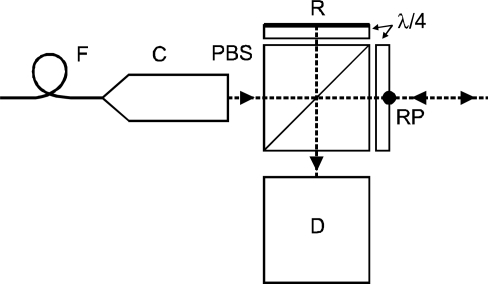
Schematics of the single-pass interferometer with a plane mirror reflector. PBS: polarizing beam splitter, C: collimator, λ/4: retardation plate, D: detection unit, F: optical fiber, R: reflective surface, RP: reference point.

The interferometer unit for this experiment was chosen to be in a simple single-beam setup where both the measuring arm and the reference arm are passing through a polarizing beamsplitter and retardation plate. The configuration of the interferometric unit is depicted in Figure 4. A more traditional configuration of a plane-mirror interferometer with two-beam improvement of resolution was avoided due to the imbalance in the length of beam paths in the optical components. Further, due to a more complex structure it seemed to be more difficult to avoid all additional shifts associated with thermal expansion. The thermal expansions of the beamsplitter and retardation plates in both interferometer arms can compensate each other provided the reference point (RP) of the whole unit is on the front plane of the retardation plate. The point RP represents a point of fixing the interferometric unit to the baseplate.

Mechanical design of the setup is a simple set of optical parts fixed to a baseplate of Zerodur ceramics including the positioning of the movable measuring mirror which is in a 100 mm range through a precision linear stage with stepping motor drive. The interferometric signal detection is based upon the polarization-optical homodyne approach with transfer of the interference state into sine-cosine signals by means of a retardation plate. This detection system—though more complicated compared to heterodyne electronic systems—operates with single-frequency lasers and is easy to implement when a tunable laser source is needed. The design of the interferometric units follows the needs of this configuration. The body is made of titanium alloy with thermal expansion coefficient close to the one of the polarizing beamsplitter SF 14. The interference optics is fixed to the baseplate with the holder with a ball at the reference point thereby eliminating the influence of its thermal expansion.

The single-pass arrangement of the interferometer operates in a simple fringe-counting regime on λ/2 resolution. Quadrature analog signals are digitized (12-bit DACs) and processed in a DSP unit with fringe counting embedded in the hardware and actual phase is calculated in a DSP processor. Hardware as well as software has been developed at our institute. The processing of the interference signal includes a linearization method [40,41]. Linearity errors of the interferometers were at the 3–4 nm level. After the evaluation of the interference phase the wavelength resolution of λ/2048 results in a resolution of 300 pm for 633 nm wavelength. The linearization technique implemented here reduces the linearity error on the level of a single discrete LSB (least significant bit)—the resolution of the interferometer. Similar techniques have been presented by other groups and in various versions are widely used in nanometrology [42–44].

The setup was assembled on an antivibration optical table with active air damping in our underground laboratory. The level of vibrations influencing noise in the interferometric signal was kept well below 1 nm when the setup was enclosed in the thermal control box. An increased level of vibrations was introduced by water flow during the heating process. This additional noise did not surpass several nanometers, thus its influence on the measurement results was negligible.

### 3.2. Alignment

**Figure 5.**
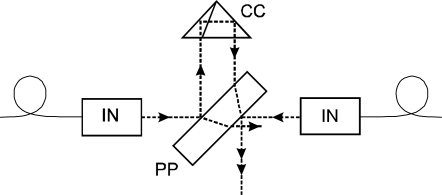
Alignment configuration for parallelism of two couterpropagating beams. IN: interferometer unit, CC: corner cube, PP: plane-parallel plate.

In order to prove the principle in comparison with a traditional indirect evaluation of the refractive index through the Edlen formula within the quite narrow tuning range of the He-Ne laser, both interferometers in the proposed countermeasuring configuration should operate without cosine and Abbe errors. With the 100 mm displacement range of the stage with the moving mirror, the whole effect of tracking of the refractive index drift over the temperature change below 1 K is 100 nm. Errors caused by angle misalignment should thus be on a few nm scale to get results that would be a proof with acceptable reliability. Predominantly the alignment of the two counterpropagating beams was critical. We designed a configuration with the help of a precise corner-cube reflector and a plane-parallel plate with semireflecting surfaces on both planes to get the beams in-line (Figure 5). The reflector and plane-parallel plate are both custom made optical components with high precision of surfaces and angle errors. The error of beam and surface parallelism were measured and were below 5 arc seconds.

In the configuration in Figure 5, with the moving mirror removed, the beams were made parallel over a large distance with precision below 10 arc seconds. Perpendicularity of the moving mirror was adjusted in a similar way combining the reflected beam from the mirror with a reference beam. The errors arising from misalignment were thus kept below the nm level as anticipated.

## 4. Recording of the Refractive Index Drift

The experiment presented here was performed in a static regime with the movable mirror in a fixed position in the center position between the two interferometric units. The temperature-controlled box served here for varying the refractive index of air through temperature. We recorded the key parameters of atmosphere inside—temperature, pressure, humidity and the content of CO_2_ and calculated the value of refractive index through Edlen formula. Especially the temperature sensors were placed as close to the laser beam as possible to get results corresponding to the interferometric measurement. Together with heating the air in the box through the walls from all sides, thermal gradients were reduced. The thermal drift of the refractive index resulted in varying of the wavelength and varying of the output from both interferometers. We added a laser frequency control following a zero sum value of the two countermeasuring interferometers. Servo-control of the laser frequency kept the wavelength of the laser constant on air and produced optical frequency drift. In our experiment we measured the laser optical frequency during the process of temperature (refractive index) drift and simultaneously recorded the value of the changing refractive index evaluated indirectly. The proof of the concept was based on comparison of these two values.

**Figure 6.**
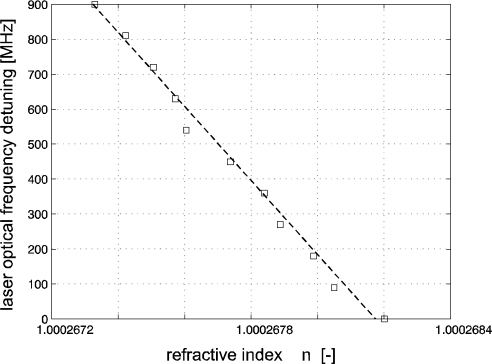
Recording of the refractive index drift and associated laser frequency tuning following the zero sum of the both interferometers output.

The range of the refractive index shift was limited by the mode-hop free tuning range of the He-Ne laser source—approximately 1 K. Overall beam length in measuring paths (both interferometers, both directions, useful motion of the mirror plus some dead length) was 321 mm. The overall relative change in the optical path expressed through the laser tuning over 900 MHz was 9.2 × 10^−7^. We used as the value for comparison the change of the refractive index 8.7 × 10^−7^ evaluated through indirect measurements of the parameters of atmosphere and calculation using the Edlen formula. The recording of the laser frequency detuning and the refractive index drift is in Figure 6. The recording is a dependence of the indirectly measured values of the refractive index of air together with the laser frequency detuning (squares) and the linear fit (dashed line). Deviations of the measured values from linear dependence are caused by limited resolution of the sensors producing 1 LSB steps. It can be clearly seen that the drift of the laser frequency follows linearly the drift of refractive index. The technique of laser frequency tracking of the constant wavelength within this range shows in our arrangement an agreement of 5 × 10^−8^, where the sources of differences are suggested to be primarily differences in time constants of thermal expansion of the mechanical components.

## 5. Conclusions

The key concept of the technique has been proven in our experiments employing the countermeasuring configuration of interferometers referenced to a baseplate made of low thermal drift material. In a static regime—where the bi-directional mirror was in a fixed position and the laser tuning followed the drift of refractive index—the wavelength was kept constant and the whole assembly could be seen as a sort of a standing wave interferometer. The experimental results are in a good agreement with the level of 5 × 10^−8^, which is generally considered close to the limit of resolution of laboratory refractometers. This is not limited by the precision of measurement but by gradients of refractive index (primarily due to gradients of temperature) between the measuring beam paths and by the microturbulations of air. Elimination or at least strong suppression of these effects represents the major contribution of the approach presented here.

In configurations where the laser interferometer(s) measure the displacement of the defined object (such as the movable table of a microscope) over a specified range it seems to be feasible to implement the compensation of refractive air fluctuations. The increase of complexity and cost of two-directional measurement can be considered relatively small compared to the sophisticated positioning stages with angle and motion control. The application of such systems is to be directed into primary nanometrology in combination with tools such as local probe microscopy and related techniques.
